# High‐Frequency Ultrasound Evaluation of Cutaneous Surgical Wound Healing: An Outpatient Experience

**DOI:** 10.1111/wrr.70136

**Published:** 2026-02-27

**Authors:** Anna Russo, Vittorio Patanè, Lucrezia Bucciero, Maria Cristina Pezzella, Mario Brunese, Francesco Stanzione, Mario Faenza, Alfonso Reginelli

**Affiliations:** ^1^ Department of Precision Medicine University of Campania “Luigi Vanvitelli” Naples Italy; ^2^ UOC di Chirurgia Generale, D'urgenza e di Endocrinochirurgia, Pineta Grande Hospital Caserta Italy; ^3^ Multidisciplinary Department of Medical, Surgical and Dental Sciences, Plastic Surgery Unit University of Campania “Luigi Vanvitelli” Naples Italy

**Keywords:** collagen remodelling, doppler imaging, high‐frequency ultrasound, skin regeneration, surgical scars, wound healing

## Abstract

High‐frequency ultrasound (HFUS) allows non‐invasive visualization of skin microarchitecture, offering quantitative assessment of dermal composition and vascularity, but its systematic use to track temporal changes in postoperative wound healing is still limited. This study aimed to describe and validate HFUS morphologic and vascular features corresponding to the biological phases of cutaneous surgical wound healing. A total of 730 patients who underwent surgical excision of skin lesions were evaluated at different postoperative intervals using high‐ and ultra‐high‐frequency ultrasound (48–70 MHz). Dermal thickness, echogenicity and vascularity were analysed with B‐mode and colour Doppler imaging through quantitative and semi‐quantitative methods and reproducibility was assessed using intraclass correlation coefficients (ICC) and Cohen's κ statistics. Cross‐sectional analysis demonstrated a progressive structural and vascular evolution consistent with canonical healing phases: dermal thickness decreased from 2.45 ± 0.38 mm at T0 to 1.58 ± 0.21 mm at T4, while echogenicity increased from 0.5 [0–1] to 2.5 [2, 3], reflecting collagen compaction and maturation. Vascularity peaked at T2 (2.2 ± 0.5) and declined to 0.8 ± 0.3 by T4, paralleling the regression of angiogenesis. Measurement reproducibility was excellent (ICC = 0.91; κ = 0.82). HFUS morphologic patterns closely mirrored the biological sequence from inflammatory oedema through granulation and fibroplasia to collagen remodelling, providing real‐time in vivo correlates of tissue repair. These findings support HFUS as a reliable, quantitative and reproducible tool for monitoring postoperative wound healing and as a potential imaging biomarker framework for early detection of abnormal scar evolution.

## 1 Introduction

Wound healing is a complex, multifactorial biological process essential for restoring the structural and functional integrity of the skin after injury or surgery [1, 2, 3].

In dermatologic oncology, where surgical excision of primary tumours such as melanoma or atypical melanocytic lesions remains a cornerstone of treatment, optimal wound repair is critical not only for functional and aesthetic recovery but also for ensuring reliable oncologic follow‐up [4, 5].

The skin, as a highly vascularized and immunologically active organ, responds to injury through a tightly regulated cascade of events encompassing overlapping phases of haemostasis and inflammation, proliferation and remodelling [6, 7, 8, 9, 10].

During the inflammatory phase, which begins within minutes to hours after tissue damage, vascular constriction is followed by vasodilation, plasma extravasation and recruitment of neutrophils, monocytes and other inflammatory cells [11, 12].

The dermis in this stage appears oedematous and poorly structured, rich in pro‐inflammatory cytokines such as IL‐1, TNF‐α and prostaglandins [13, 14].

The subsequent proliferative phase involves fibroblast activation, angiogenesis, and deposition of extracellular matrix (ECM) components such as type III collagen and proteoglycans, forming a transient granulation tissue [15, 16, 17, 18].

Finally, in the remodelling phase, myofibroblasts reorganize the ECM, leading to tissue contraction, replacement of type III with type I collagen and the re‐establishment of dermal architecture [19, 20, 21].

Recent advances in regenerative and molecular biology have expanded this classical tri‐phasic model [22, 23, 24].

Monitoring this continuum is particularly relevant in oncologic dermatology, where early recognition of abnormal healing or recurrence is vital [25, 26, 27, 28].

However, conventional follow‐up tools—visual inspection, dermoscopy and digital photography—are restricted to surface assessment and lack the capacity to visualize subsurface remodelling.

Histopathology remains the gold standard for characterizing tissue repair but is invasive, impractical for serial evaluations, and ethically unsuitable for healthy postoperative scars.

High‐frequency ultrasound (HFUS) offers a non‐invasive, reproducible, and dynamic alternative, capable of depicting dermal and superficial subcutaneous structures with micrometric resolution [29].

Devices operating above 20 MHz, and particularly ultra‐high‐frequency transducers (48–70 MHz), achieve axial resolutions of 30–60 μm and penetration depths of 3–4 mm, ideal for visualizing epidermal and dermal layers as well as subtle inflammatory and fibrotic changes [30, 31].

Although HFUS is well established in tumour imaging, inflammatory dermatoses and cosmetic assessment, its application to the systematic evaluation of surgical wound healing remains underexplored [32, 33]. Surgical wounds healing by first intention offer an ideal model for standardizing ultrasound evaluation, as they follow a predictable biological sequence with minimal confounding inflammation. This makes them particularly suitable for mapping the progressive physiopathologic transitions of healing in vivo. In this context, HFUS has the potential to provide a phase‐correlated, real‐time representation of cutaneous repair.

### 1.1 Study Rationale and Objective

Building on the routine activity of a dedicated high‐ and ultra‐high‐frequency ultrasound outpatient clinic, this study sought to validate the application of HFUS for monitoring postoperative wound healing.

Rather than following individual patients longitudinally, we analysed 730 HFUS examinations performed at different time points of the healing process, thereby reconstructing a comprehensive, cross‐sectional representation of scar maturation.

Since some patients contributed more than one examination, the total number of unique participants was lower than the total number of scans.

We hypothesized that specific HFUS morphologic and vascular features would correlate with the canonical biological phases of skin repair—inflammation, proliferation, and remodelling—and that these imaging biomarkers could serve as reproducible indicators of physiological or pathological healing trajectories.

## 2 Methods

The study's protocol was approved by the local ethics committee at the University Hospital of Campania ‘L. Vanvitelli’ and AORN ‘Ospedale dei Colli’, Naples, Prot. N. 36,255/i/2023 (15 November 2023). Given the retrospective nature of the study, the requirement for informed consent was waived by the local ethics committee. Informed consent for publication of anonymized clinical images was obtained. The study adhered to the principles outlined in the Declaration of Helsinki regarding experimentation involving human subjects.

### 2.1 Study Design and Patient Population

This study represents a prospective observational analysis derived from the routine activity of a dedicated high‐ and ultra‐high‐frequency ultrasound (HFUS/UHFUS) outpatient clinic focused on the evaluation of postoperative cutaneous wound healing at the Radiology Department of University Hospital “Luigi Vanvitelli” in Naples, Italy.

Rather than a predefined cohort followed longitudinally, the present work systematizes the cumulative clinical and imaging experience acquired over a one‐year period, with the aim of verifying and validating the applicability of HFUS for monitoring the physiological progression of surgical wound repair.

A total of 730 HFUS examinations were performed between January and December 2024 following excisional or incisional surgery for benign, premalignant or malignant cutaneous lesions.

These examinations corresponded to different stages of postoperative healing, and while some patients underwent imaging at multiple time points, the total number of unique patients was lower than 730.

Standardized HFUS imaging was performed in all cases using linear probes ranging from 48 to 70 MHz.

Each examination was assigned to a specific stage of wound healing according to the timing of the clinical presentation.

The distribution of examinations across the different biological phases of repair was as follows:

- 36 within 24 h after surgery (T0, acute inflammatory phase);
- 66 at 7 days (T1, late inflammatory/early proliferative phase);
- 64 at 14–28 days (T2, proliferative phase);
- 110 at 30 days (T3, fibrovascular granulation/early remodelling phase);
- 170 at 90 days (T4, active remodelling phase);
- 160 at 12 months (T5, late remodelling/mature scar);
- 124 at 24 months (T6, stabilized scar).

Although individual patients were not followed longitudinally through all stages, and some contributed to more than one time point, this cross‐sectional dataset provided a continuous and representative depiction of the healing spectrum, from early inflammation to complete remodelling.

The study was conducted in accordance with the principles of the Declaration of Helsinki. All participants provided informed consent for ultrasound imaging and for the anonymous use of their images and data for research and educational purposes.

### 2.2 Ultrasonographic Protocol

All examinations were carried out in the same facility using a Vevo 3100 system (FUJIFILM VisualSonics, Toronto, Canada) equipped with a 48–70 MHz linear transducer.

This configuration provides an axial resolution of 30–60 μm and a penetration depth of approximately 3–4 mm, suitable for visualizing the epidermis, dermis, and superficial subcutis.

A single experienced operator, with dedicated training in dermatologic and scar ultrasonography, performed all scans to ensure technical uniformity.

Examinations were obtained in both longitudinal and transverse orientations depending on wound location. A thin layer of coupling gel—and, when necessary, a stand‐off pad—was applied to optimize acoustic contact with superficial structures.

### 2.3 Imaging Timeline and Parameters

Patients were evaluated at different stages of healing, corresponding to the seven standardized temporal windows (T0–T6), allowing a cross‐sectional reconstruction of the wound‐healing continuum.

At each time point, static B‐mode and, when feasible, colour Doppler images were obtained to assess microvascularity.

Observations focused on dermal thickness, echogenicity, structural heterogeneity, vascular signal intensity, and demarcation of the dermo‐epidermal junction.

Echogenicity and heterogeneity were classified semiquantitatively on a 0–3 scale, where 0 = normal appearance and 3 = maximum deviation from baseline morphology.

### 2.4 Quality Control and Standardisation

Because all examinations were conducted by a single operator following a predefined acquisition protocol, intraoperator consistency was prioritized over interobserver validation.

To verify reproducibility, 10% of scans were re‐evaluated after 1 week by the same operator, and results were compared using intraclass correlation coefficients (ICC).

This single‐operator model aligns with prior literature reporting high reproducibility of HFUS metrics under standardized acquisition conditions [34, 35].

### 2.5 Statistical Analysis

Quantitative parameters (e.g., dermal thickness) are expressed as mean ± standard deviation (SD), while semiquantitative variables (echogenicity and heterogeneity) are presented as median ± interquartile range (IQR).

Although the study was primarily descriptive, the collected data were analysed to identify consistent morphologic patterns across time points and plausible correlations between ultrasonographic parameters and the underlying physiopathological mechanisms of wound healing.

Comparisons across time points were performed on independent patient subgroups.

Particular attention was given to the temporal evolution of dermal echogenicity, vascular signal distribution, and tissue organization, interpreted in light of the canonical phases of inflammation, proliferation, and remodelling described in current wound‐healing biology.

## 3 Results

**FIGURE 1:**
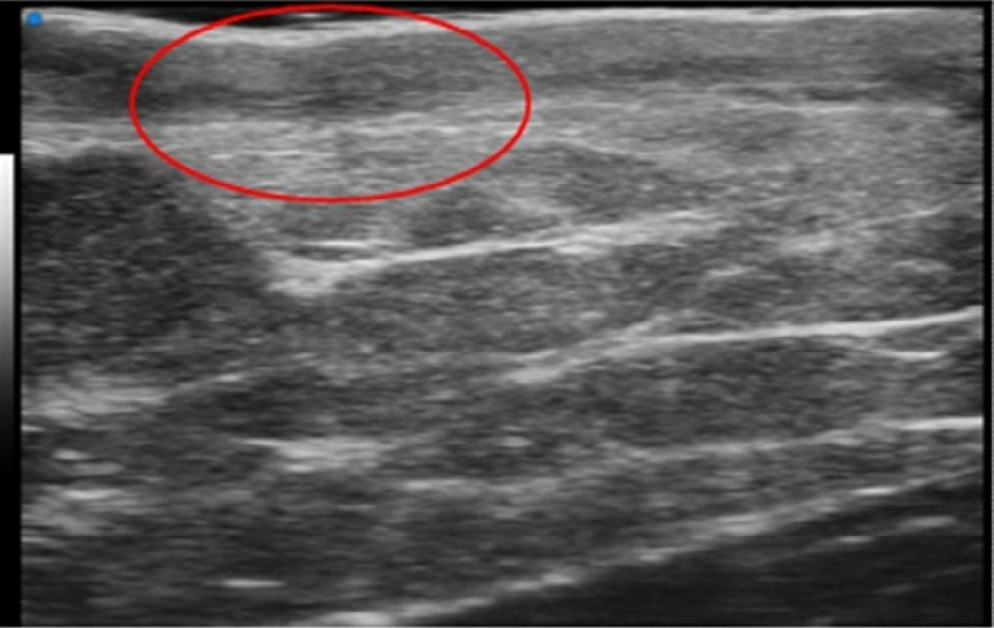
Anatomical high‐frequency ultrasound (HFUS) appearance of the skin and subepidermal fibrotic layer. Representative HFUS image illustrating the anatomical organization of the skin and the subepidermal region involved in postoperative scar formation. The superficial hyperechoic band corresponds to the epidermis, while the underlying hypoechoic dermis exhibits organized fibrous tissue deposition (highlighted by the red circle) consistent with early remodelling after surgical excision.

**FIGURE 2:**
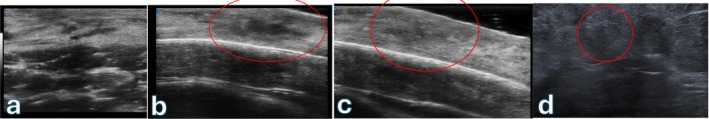
Representative ultra–high‐frequency ultrasound (UHFUS; 48–70 MHz) B‐mode images illustrating scar healing at different stages of maturation. (a) Early‐stage healing with dermal thickening and reduced structural definition. (b‐c) Intermediate stage with a heterogeneous (‘mosaic’) echotexture consistent with active tissue remodelling; the red circle highlights the main focal alteration. (c) Later‐stage remodelling with progressively more organized dermal architecture; the red circle marks the residual area of altered echogenicity/texture.

**FIGURE 3:**
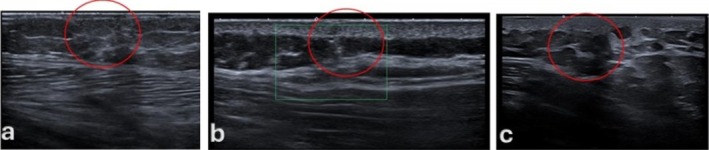
Representative ultra–high‐frequency ultrasound (UHFUS; 48–70 MHz) B‐mode images showing scar‐related dermal alterations across different examinations. The red circles highlight the main area of altered echotexture (typically heterogeneous and/or relatively hypoechoic compared with adjacent dermis), consistent with ongoing scar remodelling.

Throughout the study period, most patients experienced healing by first intention, with no clinically relevant complications such as infection or wound dehiscence; when present, atypical patterns (e.g., seroma, hematoma and keloid) are detailed in Section 3. High‐frequency ultrasound (HFUS) provided detailed and reproducible imaging of the surgical sites across temporal stages, allowing visualization of distinct tissue morphologies corresponding to the inflammatory, proliferative and remodelling phases of wound healing (Figures 1, 2, 3).

A summary of the main HFUS morphologic findings and their correspondence with biological healing phases is provided in Table 1. These imaging findings were then interpreted in light of current knowledge of the molecular and cellular mechanisms underlying tissue repair.

**TABLE 1:** Summary of HFUS morphologic features observed at sequential postoperative time points (T0–T4) and their correspondence with the canonical biological phases of wound healing.

Time point	Postoperative period	Predominant biological phase	Main HFUS morophologic findings	Physiopathologic correlation
T0	24–48 h	Early Inflammation	Marked dermal thickening; diffuse hypoechogenicity; blurred dermo‐epidermal junction; minimal or absent Doppler signal.	Interstitial oedema and vascular leakage due to acute inflammation; early neutrophilic infiltration and capillary shutdown.
T1	7 days	Ongoing inflammation/onset of proliferation	Persistent dermal swelling; heterogeneous low‐level echoes; beginning of perivascular Doppler activity.	Transition between inflammatory and proliferative phases; macrophage recruitment and early angiogenesis.
T2	14 days	Active proliferation (granulation phase)	Increased dermal thickness; irregular mosaic echotexture with multiple iso‐ and hypoechoic foci; marked Doppler vascularity.	Fibroblast and endothelial proliferation; ECM deposition (type III collagen); intense neoangiogenesis (VEGF, TGF‐β).
T3	30 days	Late proliferation/early remodelling	Partial homogenization of dermal structure; decreasing vascularity; reappearance of dermo‐epidermal interface.	Progressive collagen maturation and alignment; resolution of inflammatory activity.
T4	90 days	Remodelling/maturation	Homogeneous hyperechoic dermis; well‐defined dermo‐epidermal line; stabilized dermal thickness; minimal vascular signal.	Predominant type I collagen deposition; reduction of fibroblast activity and vascular regression; scar stabilization.

*Note:* The table illustrates the progressive transition from hypoechoic, oedematous dermal patterns in the early inflammatory phase to heterogeneous, vascularized granulation tissue during proliferation, and finally to homogeneous, hyperechoic scar morphology during the remodelling stage. Each temporal stage reflects a distinct set of cellular and molecular events – ranging from vascular leakage and immune infiltration to fibroblast activation, extracellular matrix deposition and collagen realignment – captured non‐invasively through HFUS imaging.

**FIGURE 4:**
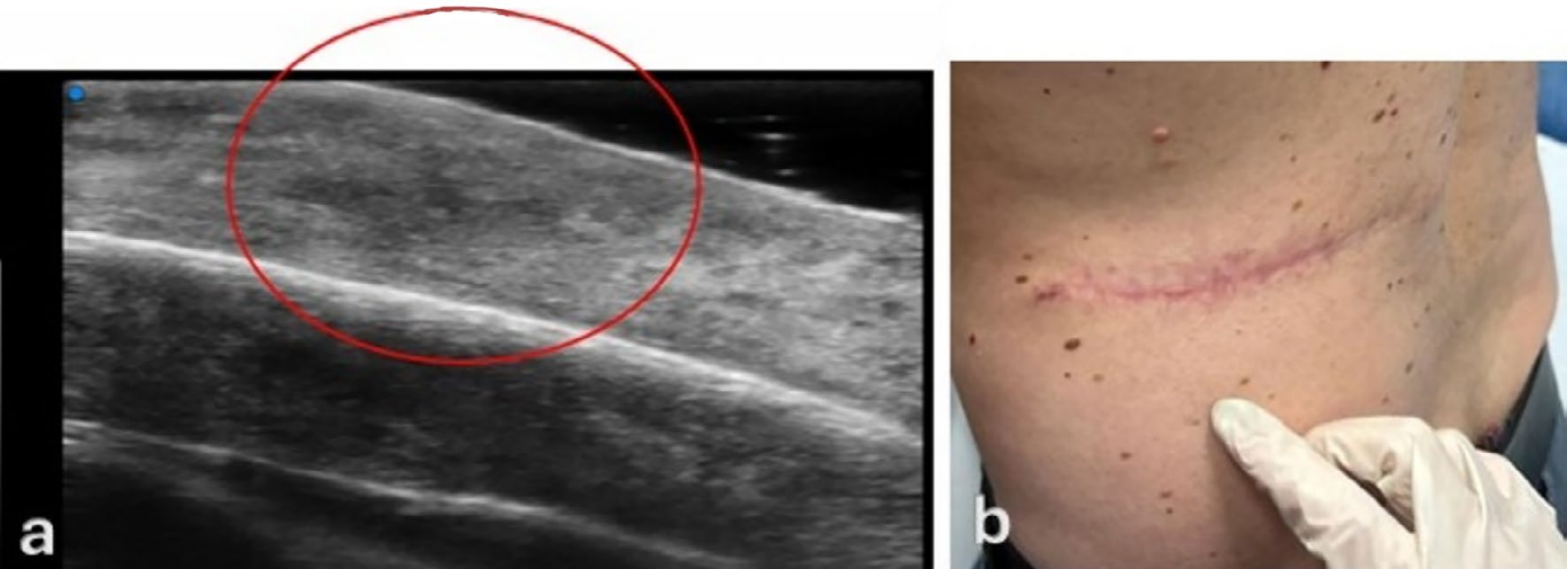
Temporal evolution of HFUS parameters during surgical wound healing. The line graph illustrates the dynamic progression of three key imaging biomarkers—dermal thickness, echogenicity, and vascularity—measured at sequential postoperative intervals (T0–T4). A progressive decrease in dermal thickness reflects resolution of oedema and tissue compaction, while the steady increase in echogenicity corresponds to collagen maturation and extracellular matrix organization. The vascularity curve demonstrates a transient peak at day 14 (T2), consistent with the angiogenic activity of the proliferative phase, followed by a marked decline during remodelling. Together, these parameters depict the physiologic transition from inflammation to proliferation and remodelling, confirming the potential of HFUS as a non‐invasive biomarker of wound‐healing dynamics.

Quantitative analysis showed a progressive and statistically significant reduction in dermal thickness, consistent with the resolution of oedema and the progressive maturation of scar tissue. Cross‐sectional analysis revealed a consistent trend of decreasing dermal thickness across progressive healing stages, from 2.45 ± 0.38 mm at T0 to 1.58 ± 0.21 mm at T4. Semi‐quantitative analysis of echogenicity revealed a complementary pattern, with median values increasing from 0.5 [0–1] at T0 to 2.5 [2, 3] at T4, reflecting the progressive rise in dermal reflectivity as collagen fibres became denser and more organized. Colour Doppler assessment demonstrated that vascular signal intensity peaked at T2 (mean 2.2 ± 0.5) and declined markedly by T4 (0.8 ± 0.3), in line with the expected reduction in angiogenic activity during late healing. Intra‐operator reproducibility for dermal thickness measurements was excellent (ICC = 0.91; 95% CI 0.86–0.95), confirming high measurement reliability and stability of acquisition parameters. Figure 4 shows the temporal evolution of the three principal HFUS parameters—dermal thickness, echogenicity, and vascularity—demonstrating their complementary trends across the wound‐healing timeline.

A summary of quantitative and semi‐quantitative HFUS parameters across all postoperative time points is provided in Table 2.

**TABLE 2:** Quantitative and semi‐quantitative parameters derived from HFUS imaging at sequential postoperative intervals (T0–T4).

Time point	Postoperative interval	Mean dermal thickness (mm ± SD)	Echogenicity score (median [IQR] ± SD, 0–3)	Vascularity score (mean ± SD, 0–3)	Predominant healing phase
T0	24–48 h	2.45 ± 0.38	0.5 [0–1]	0.3 ± 0.2	Early inflammation
T1	7 days	2.32 ± 0.33	1.0 [0.5–1.5]	0.8 ± 0.4	Late inflammation/onset of proliferation
T2	14 days	1.98 ± 0.29	1.5 [1–2]	2.2 ± 0.5	Active proliferation (granulation)
T3	30 days	1.72 ± 0.26	2.0 [1.5–2.5]	1.4 ± 0.4	Transition to remodelling
T4	90 days	1.58 ± 0.21	2.5 [2–3]	0.8 ± 0.3	Remodelling/maturation

*Note:* A progressive decrease in dermal thickness was observed across stages, consistent with oedema resolution and scar compaction, while echogenicity increased steadily in parallel with collagen maturation. Vascular signal intensity peaked during the proliferative phase (T2) and decreased thereafter, reflecting the expected regression of angiogenesis. These imaging metrics collectively delineate the dynamic transition from inflammation to remodelling and provide potential objective biomarkers of wound‐healing progression.

During the early postoperative period (T0–T1; 24–48 h to 7 days), HFUS imaging displayed marked dermal thickening, diffuse hypoechogenicity, and loss of dermo‐epidermal definition, findings consistent with interstitial oedema and capillary permeability associated with acute inflammation. Doppler flow was minimal or absent, reflecting transient vascular constriction and stasis. These morphologic changes correspond to the biological milieu dominated by neutrophilic infiltration, macrophage recruitment, and cytokine‐driven vascular leakage. At this stage, the ultrasound depiction of an expanded, low‐echogenic dermis served as a morphologic surrogate for inflammatory oedema and early immune activation.

**FIGURE 5:**
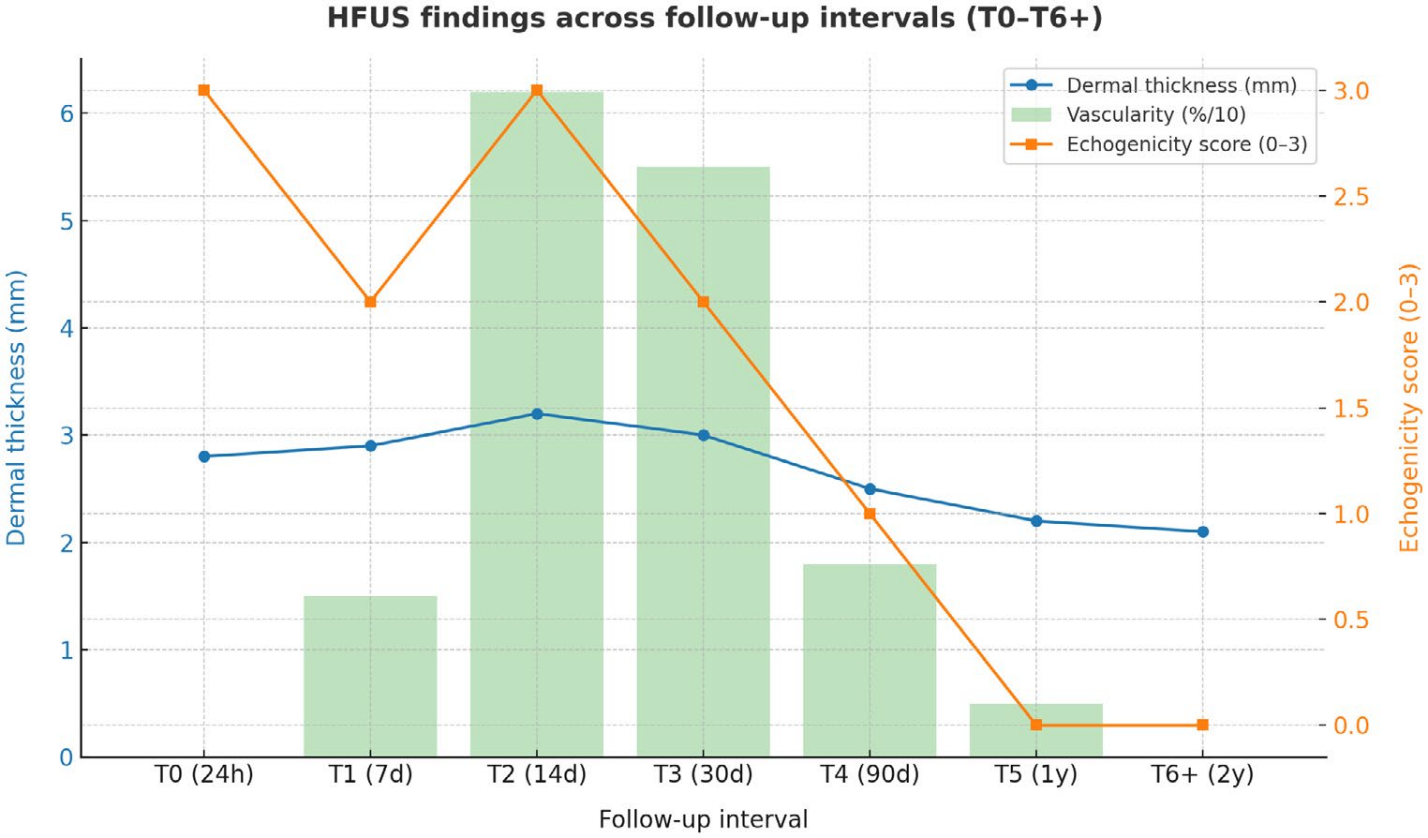
Clinical and imaging correlation of the fibrovascular granulation stage, representing the transition from the proliferative to the early remodelling phase. The HFUS image (a) shows a heterogeneous, predominantly hyperechoic dermis corresponding to fibroblast proliferation and early collagen type I deposition (red circle). The clinical photograph (b) demonstrates a postoperative scar at one week, with visible re‐epithelialization and early tissue integration. This phase marks the peak of fibroblast activity and the transition toward structural maturation of the scar.

Between 14 and 30 days post‐surgery (T2–T3)—the proliferative phase—HFUS images revealed increased dermal heterogeneity with a characteristic mosaic pattern composed of mixed iso‐ and hypoechoic areas, often accompanied by intense Doppler vascularization. Dermal thickness remained elevated but progressively declined toward T3. These findings mirror the histologic events of fibroblast activation, extracellular‐matrix deposition, and angiogenesis, with evolving granulation tissue bridging the wound gap. The echotextural variability visualized by HFUS reflects the spatial interplay between proliferating fibroblasts, immature collagen bundles and neovascular networks. This stage corresponds to the peak of tissue biosynthetic activity, driven by the combined effects of growth factors such as VEGF and TGF‐β, as well as mechanical cues that modulate fibroblast differentiation and matrix organization. Figure 5 illustrates the fibrovascular granulation stage, corresponding to the transition between the proliferative and early remodelling phases, where fibroblast proliferation peaks and collagen type I deposition begins.

By day 90 (T4), HFUS demonstrated a clear transition to homogeneous, hyperechoic dermal patterns with a re‐established dermo‐epidermal interface and a stable dermal thickness comparable to surrounding tissue. Vascular signals were scarce or absent, consistent with completion of the remodelling and maturation phase. At this stage, the scar exhibited the acoustic characteristics of aligned type I collagen, reduced cellularity and decreased water content. The progressive increase in echogenicity and restoration of dermal stratification were interpreted as ultrasound correlates of collagen realignment and extracellular‐matrix consolidation, marking the shift from an active reparative to a stable fibrotic state.

**FIGURE 6:**
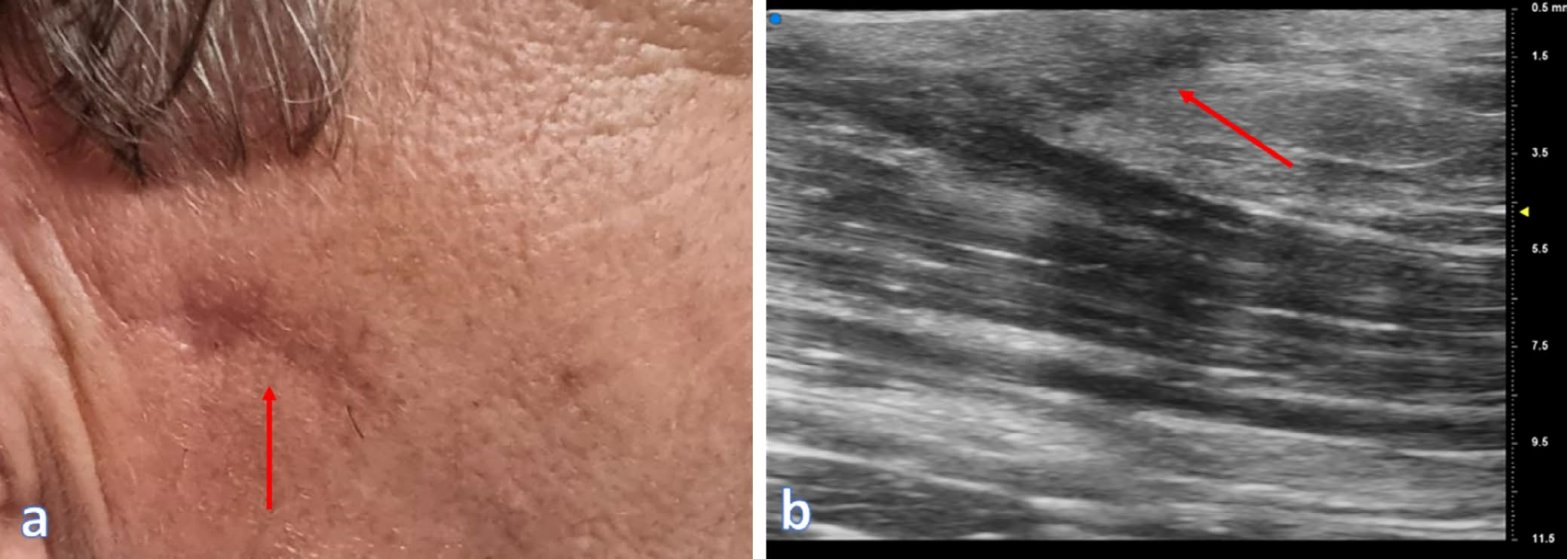
Clinical and HFUS correlation in the final remodelling phase of a postoperative scar. Panel (a) shows the clinical appearance of a mature, normotrophic scar on the malar region, marked by mild erythema but full re‐epithelialization. Panel (b) presents the corresponding HFUS image, demonstrating a well‐organized, hyperechoic dermal band with restored layering, consistent with collagen type I alignment and reduced vascularity. The correspondence between clinical and imaging findings confirms complete remodelling and structural stabilization of the scar.

Figure 6 shows the clinical and ultrasound appearance of a mature postoperative scar, illustrating the correspondence between cutaneous morphology and subepidermal organization after complete collagen realignment.

**FIGURE 7:**
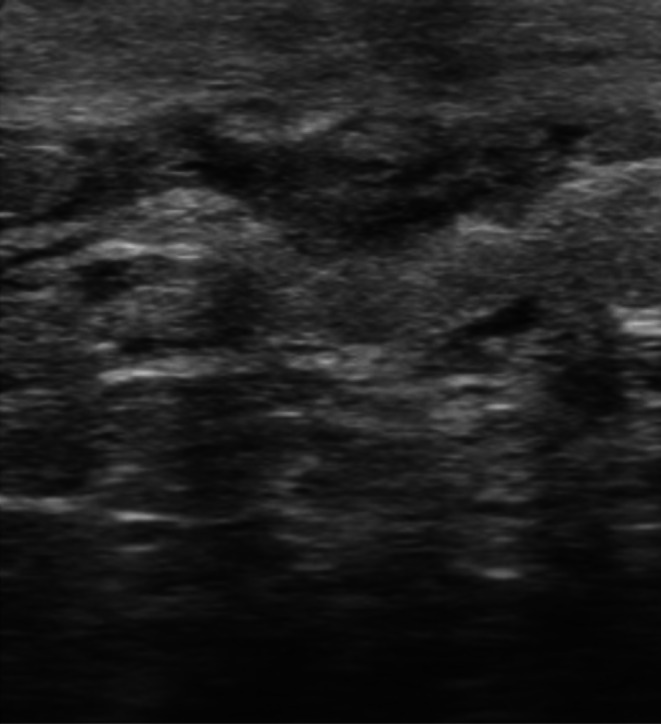
HFUS image showing fibroblastic proliferation and hyalinized collagen deposition during the proliferative phase. Representative B‐mode HFUS image acquired during the proliferative phase (T2–T3). The image shows fibroblastic proliferation with a nodular growth pattern and fascicles of hyalinized collagen, appearing iso‐ to hyperechoic due to increased reflection from compact collagen fibres. This echotextural pattern corresponds to the transition between active granulation and early remodelling, characterized histologically by fibroblast activation, extracellular matrix deposition, and the onset of collagen cross‐linking.

A representative high‐resolution HFUS image illustrating the fibroblastic proliferative pattern with hyalinized collagen deposition is shown in Figure 7.

These temporal imaging patterns align closely with the current understanding of wound‐healing biology. Recent models emphasize the interplay of mechanotransduction pathways, including YAP/TAZ and TGF‐β, that govern fibroblast activation and extracellular‐matrix remodelling. While these molecular mechanisms were not directly investigated in the present study, the sequential HFUS morphologies observed—decreasing dermal thickness, increasing echogenicity, and resolving vascularity—can be plausibly interpreted as their non‐invasive phenotypic counterparts, translating microscopic processes into measurable imaging signatures.

The semi‐quantitative scoring system (0–3 for oedema, echogenicity and fibrosis) demonstrated excellent interobserver agreement between two independent evaluators (Cohen's κ = 0.82; 95% CI 0.75–0.89), confirming the robustness and reproducibility of qualitative and semi‐quantitative HFUS interpretation.

Overall, both numerical data and imaging morphology delineated a consistent and physiologically grounded evolution across time points in which HFUS parameters accurately reflected the structural and cellular dynamics of wound repair.

In summary, HFUS effectively captured the progressive morphologic, vascular, and compositional transformations occurring during surgical wound healing. The main imaging parameters—dermal thickness, echogenicity, and vascularity—displayed predictable temporal trends paralleling the biological sequence of inflammation, proliferation, and remodelling.

These findings underscore the potential of HFUS as a non‐invasive imaging biomarker of tissue healing, providing clinicians with real‐time insights into scar maturation and offering an objective framework for distinguishing physiological from delayed or pathological wound trajectories.

### 3.1 Atypical and Complicated Healing Patterns

Although the majority of postoperative scars followed the expected temporal sequence of inflammation, proliferation, and remodelling, a subset of cases showed deviations from the physiological trajectory, underscoring the sensitivity of HFUS in detecting divergent patterns and guiding clinical decision‐making.

#### 3.1.1 Atypical Trajectories Within the Physiological Spectrum

**FIGURE 8:**
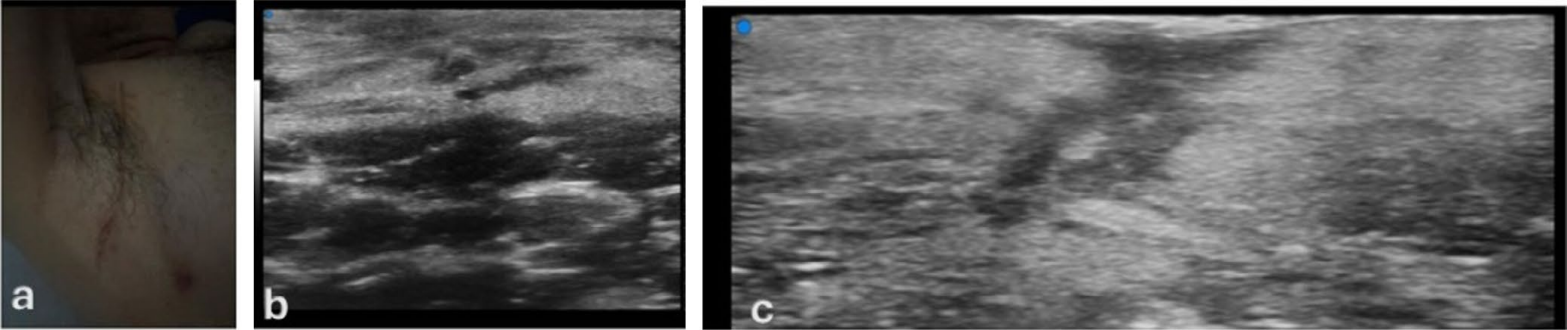
HFUS monitoring of axillary postoperative healing following lymphadenectomy. Clinical and HFUS documentation of a patient who underwent axillary lymphadenectomy. Panel (a) shows the postoperative scar in the axillary region. Panel (b) demonstrates a hypoechoic vascularized focus at day 14 (T2), compatible with inflammatory granulation tissue. By day 90 (T4), panel (c) shows a well‐organized, hyperechoic fibrotic band, consistent with complete tissue remodelling and collagen reorganization. This case exemplifies the ability of HFUS to non‐invasively track the transition from early inflammation to mature scar formation in surgically complex areas.

HFUS occasionally documented unusual evolutions that nonetheless culminated in orderly remodelling. For example, in a 66‐year‐old patient (scapular region), a multilobular hypoechoic area with peripheral vascularization at T2 evolved into a dense hyperechoic scar by T4, suggestive of exuberant granulation followed by successful consolidation. In a 58‐year‐old post‐radiotherapy patient (thoracic site), the dermis remained hypo‐ to isoechoic with poor stratification at T3, and by T4 showed marked hyperechogenicity and thinning, consistent with radiation‐induced fibrotic remodelling. A 45‐year‐old patient (axillary site post‐lymphadenectomy) presented a vascularized hypoechoic focus at T2 that evolved into a compact, hyperechoic fibrotic band by T4, confirming HFUS capability to track transient inflammatory collections and their resolution (Figure 8).

#### 3.1.2 Secondary‐Intention Healing in Irradiated Skin

**FIGURE 9:**
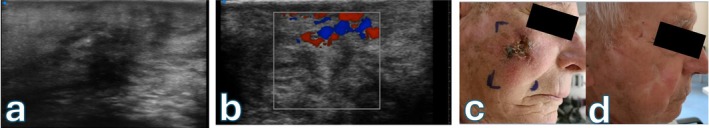
HFUS and clinical correlation in secondary‐intention healing following radiodermatitis. Panels (a) and (b): B‐mode and colour Doppler HFUS showing dermal disorganization, iso–/hypoechoic fibrosis, and persistent superficial vascular signals. Panels (c) and (d): Clinical photographs demonstrating acute radiodermatitis with ulceration and blistering (c) and partial re‐epithelialization with residual fibrosis at 1 month (d). HFUS documents secondary‐intention healing and helps differentiate persistent inflammation from organized fibrotic remodelling in irradiated skin.

A representative case of radiodermatitis following radiotherapy for basal cell carcinoma illustrated the discordant time course between surface re‐epithelialization and deeper fibrotic organization. HFUS revealed dermal disorganization with iso‐ to hypoechoic fibrotic tissue and persistent superficial vascularity, consistent with ongoing inflammation and early fibroblastic repair, while serial clinical photographs documented the gradual cutaneous recovery (Figure 9).

#### 3.1.3 Post‐Operative Complications

**FIGURE 10:**
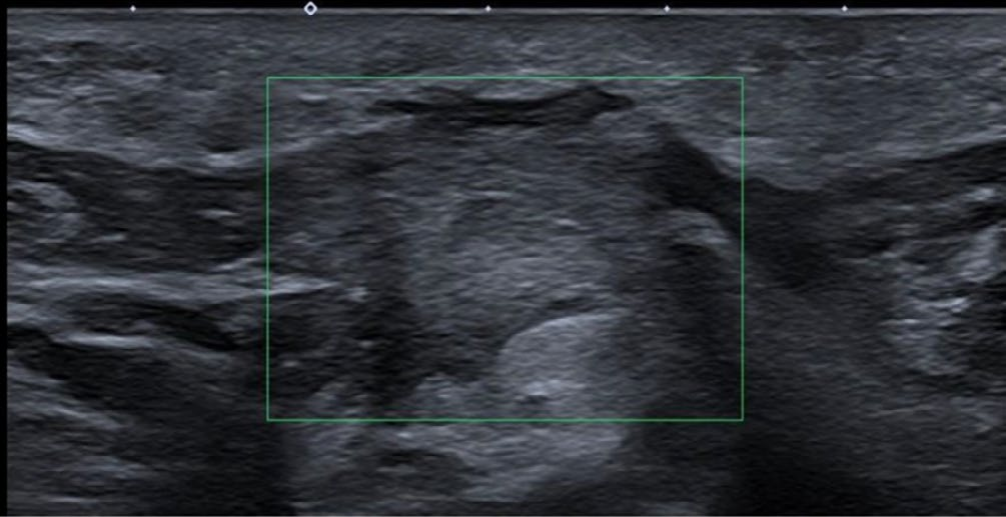
Postoperative hematoma on HFUS. The image shows an ill‐defined, heterogeneously hypoechoic area in the subcutaneous tissue, compatible with early organizational blood collection. No internal vascular signal is detected on colour Doppler, confirming non‐perfused content. Over time, these lesions typically decrease in size and echogenicity, differentiating them from abscesses or seromas by their inhomogeneous echoes and irregular contours.

HFUS also enabled the non‐invasive differentiation of early fluid collections and pathological scarring. Hematoma appeared as an ill‐defined, heterogeneously hypoechoic subdermal collection within 72 h after surgery, without internal Doppler signal, showing progressive resorption on follow‐up (Figure 10).

**FIGURE 11:**
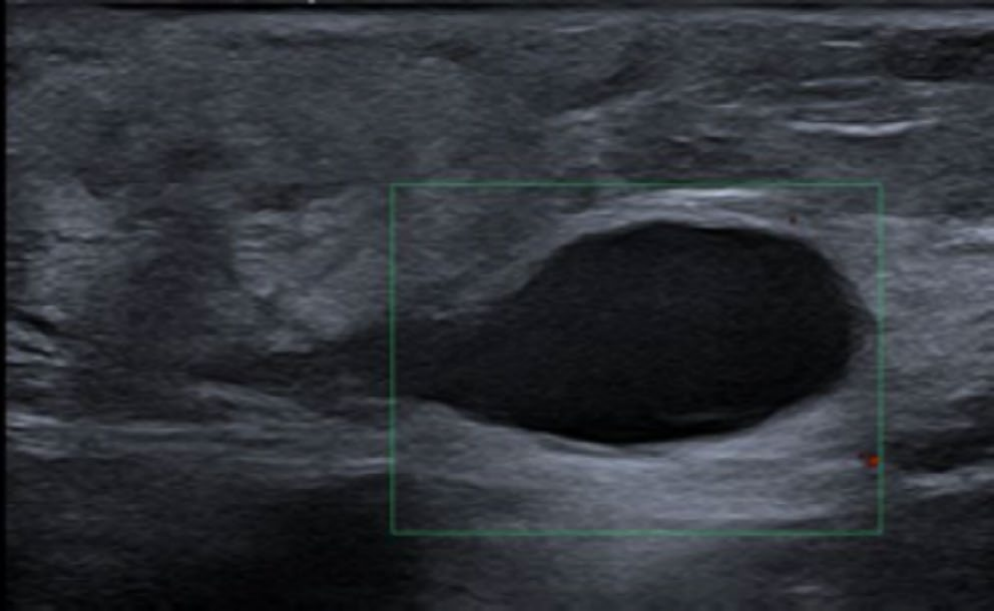
Postoperative seroma detected on HFUS. The image shows a well‐circumscribed, anechoic collection with thin, smooth walls and absent Doppler flow, consistent with sterile serous fluid in the surgical bed. Typical resolution occurs spontaneously or post US‐guided aspiration; the absence of internal echoes/flow helps differentiate it from hematoma or abscess.

Seroma presented as a well‐circumscribed, anechoic cavity with thin, smooth echogenic walls and no internal vascularity, sometimes with a fluid–fluid level, generally resolving spontaneously or after ultrasound‐guided aspiration within 2 weeks (Figure 11).

**FIGURE 12:**
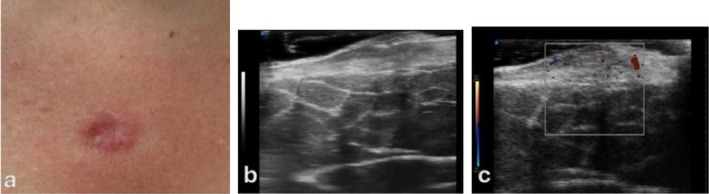
Representative HFUS findings in complicated healing patterns. (a) Clinical image of a hypertrophic/keloid scar (elevated, erythematous lesion). (b) B‐mode HFUS: Thickened, hyperechoic dermis with disrupted layering. (c) Colour Doppler: Marginal vascularity, indicating persistent cellular activity. HFUS distinguishes pathological scars from normal maturation by echogenicity, architecture and vascular profile.

Hypertrophic/keloid scar displayed marked dermal thickening, irregular hyperechoic fibrotic bands, and loss of normal stratification on B‐mode, with peripheral/marginal vascularity on Doppler indicating ongoing fibroblast/endothelial activity. This profile reflects excessive type I/III collagen deposition and dysregulated remodelling (Figure 12).

Together, these cases (Figures 9, 10, 11, 12) illustrate how HFUS complements clinical assessment by revealing subsurface structure and perfusion, enabling early recognition of deviations from physiological healing and supporting targeted management (e.g., aspiration of seroma, close surveillance of hematoma resolution or early intervention in hypertrophic/keloid evolution).

The semi‐quantitative scoring system (0–3 for oedema, echogenicity and fibrosis) demonstrated excellent interobserver agreement between two independent evaluators (Cohen's κ = 0.82; 95% CI 0.75–0.89), supporting the robustness of qualitative and semi‐quantitative image interpretation.

Overall, both the numerical data and imaging morphology delineate a consistent and physiologically grounded evolution across time points, in which HFUS features accurately reflect the structural and cellular dynamics of wound repair.

In summary, HFUS proved capable of capturing the progressive morphologic, vascular, and compositional transformations occurring during surgical wound healing. The imaging parameters—dermal thickness, echogenicity, and vascularity—showed predictable temporal trends that paralleled the biological sequence of inflammation, proliferation, and remodelling.

These results underscore the potential of HFUS as a non‐invasive biomarker of tissue healing, providing clinicians with real‐time insights into scar maturation and offering an objective framework for distinguishing physiological from delayed or pathological wound trajectories.

## 4 Discussion

Despite its cross‐sectional rather than longitudinal design, the large and temporally balanced cohort enabled the reconstruction of a continuous imaging trajectory of wound healing, effectively approximating a prospective follow‐up model. High‐frequency ultrasound (HFUS) proved to be a reliable and sensitive tool for visualizing the sequential structural and vascular transformations occurring during surgical wound healing. The morphologic evolution observed—progressive reduction in dermal thickness, increased echogenicity and dynamic modulation of vascularity—closely mirrors the canonical biological sequence of inflammation, proliferation, and remodelling. These imaging changes provide an objective, non‐invasive reflection of the cellular and molecular mechanisms underpinning tissue repair.

### 4.1 Biological Interpretation of HFUS Patterns

During the early inflammatory phase (T0–T1), HFUS depicted diffuse dermal hypoechogenicity and loss of structural definition, findings that correspond to interstitial oedema and increased vascular permeability induced by inflammatory mediators such as IL‐1 and TNF‐α. The lack of Doppler signal likely reflects transient microvascular shutdown and tissue imbibition—responses well documented in acute wound physiology [36, 37].

In the proliferative phase (T2–T3), the dermis acquired a heterogeneous, mosaic‐like echotexture with prominent vascular signals, reflecting fibroblast proliferation, angiogenesis, and extracellular matrix (ECM) deposition. The persistence of dermal thickening at this stage is consistent with active ECM synthesis and type III collagen accumulation [38, 39]. The Doppler peak observed at T2 functionally correlates with VEGF‐ and TGF‐β–driven angiogenesis, confirming that HFUS can capture not only structural but also physiological dimensions of healing [40, 41].

By the remodelling phase (T4), HFUS showed homogeneous hyperechoic dermal tissue with re‐establishment of the dermo‐epidermal junction and stable dermal thickness. This corresponds to type I collagen alignment, reduced fibroblast activity, vascular regression and dehydration of the scar—hallmarks of a mature, mechanically stable tissue [42].

These findings integrate well with current models of mechanotransduction in wound repair. The YAP/TAZ and TGF‐β pathways regulate fibroblast differentiation and collagen architecture, determining final scar organization [43, 44, 45]. The progressive increase in echogenicity and reduction in vascularity detected by HFUS likely represents imaging correlates of these mechanobiological events, confirming the role of HFUS as a phenotypic translator of molecular remodelling.

### 4.2 Integration With Existing Literature

The correlation between HFUS findings and physiological phases supports its value as a dynamic monitoring tool. Unlike static clinical inspection, HFUS provides quantitative, reproducible insight into the microstructural evolution of healing tissue, bridging histological processes and real‐time imaging.

Our results align with and expand previous reports [46, 47, 48], which documented progressive increases in echogenicity and collagen reorganization during healing. However, those studies were generally limited by smaller cohorts or single time‐point analyses. In contrast, the present work integrates imaging findings with a phase‐correlated biological framework, offering a structured interpretive model that links ultrasound patterns to the tri‐phasic biology of wound repair.

Technically, reproducibility in our cohort (ICC = 0.91) was consistent with benchmark HFUS reliability values (0.85–0.94). The combination of B‐mode and colour Doppler provided complementary structural and vascular insights, confirming the benefit of multimodal ultrasonography in dermatologic imaging.

Our results also reinforce the view that clinical inspection alone underestimates subepidermal changes. Previous work [49] reported similar discrepancies between surface appearance and deep‐tissue remodelling. The gradual increase in echogenicity and delayed restoration of the dermo‐epidermal interface seen in our series supports this observation and highlights the capacity of HFUS to detect microstructural maturation before it becomes clinically visible.

### 4.3 Clinical Implications

By quantifying parameters such as dermal thickness, echogenicity, and vascularity, HFUS provides a non‐invasive biomarker‐based framework for postoperative scar monitoring. Persistent hypoechogenicity or hypervascularity may signal delayed remodelling or pathological scarring, whereas normalization of echogenicity indicates tissue stabilization. The integration of HFUS into routine postoperative follow‐up could therefore enable personalized surveillance protocols. Patients showing early normalization may require less frequent monitoring, while those with abnormal imaging patterns could benefit from intensified observation or early therapeutic intervention. The addition of colour Doppler further expands the diagnostic scope, allowing differentiation between active granulation, inflammation or early fibrosis. Persistence of vascular signals beyond expected timelines may alert to infection, chronic inflammation, or hypertrophic evolution. This morpho‐functional approach transforms HFUS from a purely structural imaging technique into a comprehensive diagnostic and predictive modality—particularly valuable in dermatologic oncology and reconstructive surgery, where precise documentation of healing trajectories is essential.

### 4.4 Methodological and Educational Considerations

The reproducibility demonstrated in this study (ICC = 0.91) confirms the consistency of HFUS measurements under standardized acquisition protocols. This supports its use as a quantitative endpoint in clinical trials evaluating wound‐healing agents or surgical techniques. Beyond its clinical utility, HFUS also offers educational value, allowing real‐time visualization of cutaneous repair dynamics and reinforcing the conceptual understanding of healing biology among clinicians and trainees.

## 5 Limitations

This study has several limitations that should be acknowledged.

First, the cross‐sectional design precluded longitudinal follow‐up of individual patients, although the large sample size and balanced distribution across healing stages provided a representative overview of the temporal sequence. Second, the investigation was conducted in a single centre, potentially introducing selection bias related to patient demographics and wound characteristics. The population mainly consisted of individuals undergoing excision of benign or suspicious melanocytic lesions, limiting extrapolation to traumatic or chronic wounds. Third, the study design was observational and descriptive. Although strong temporal and biological correlations were identified, causality between HFUS features and histological events cannot be definitively inferred. Future research combining HFUS with histopathology, confocal microscopy, or molecular assays could validate the proposed imaging biomarkers. Fourth, while semi‐quantitative scoring of echogenicity and vascularity proved reproducible, it remains partly subjective. Despite these constraints, this study establishes a robust foundation for future phase‐correlated, multi‐parametric HFUS investigations of cutaneous wound healing.

## 6 Conclusion

High‐frequency ultrasound (HFUS) offers a unique, non‐invasive window into the dynamic architecture of postoperative wound healing. By quantifying structural and vascular parameters—dermal thickness, echogenicity and perfusion—it provides measurable imaging correlates of the biological continuum from inflammation to remodelling. The systematic correspondence between HFUS features and wound‐healing phases underscores its potential as an imaging‐based biomarker framework for objective scar assessment. HFUS not only complements clinical examination but also enables early detection of abnormal healing, paving the way for personalized management and image‐guided therapeutic interventions.

## Conflicts of Interest

The authors declare no conflicts of interest.

## Data Availability

The data that support the findings of this study are available on request from the corresponding author. The data are not publicly available due to privacy or ethical restrictions.
